# Identifying compound heterozygous variants in the *EEFSEC* gene linked to progressive cerebellar atrophy

**DOI:** 10.1186/s11689-025-09632-6

**Published:** 2025-07-12

**Authors:** Zhen Liu, Mei He, Xuan Luo, Hu Pan, Juanli Hu, Zhengqing Wan, Yin Peng, Yixiao Luo, Hua Wang, Xiao Mao

**Affiliations:** 1 Key Laboratory for Birth Defects Research and Prevention of the National Health Commission, Hunan Provincial Maternal and Child Health Care Hospital, Changsha, Hunan PR China; 2 School of Life Sciences, Central South University, Changsha, Hunan PR China; 3 Department of Medical Genetics, Hunan Provincial Maternal and Child Health Care Hospital, Changsha, Hunan PR China; 4 Hunan Province People’s Hospital, The First-Affiliated Hospital of Hunan Normal University, Changsha, Hunan PR China; 5 The Affiliated Children’s Hospital Of Xiangya School of Medicine, Central South Universityï (Hunan children’s hospital), Changsha, Hunan PR China; 6 Clinical Medical Research Center For Hereditary Birth Defects and Rare Diseases In Hunan Province, Changsha, Hunan PR China; 7 The Affiliated Children’s Hospital Of Xiangya School of Medicine, Clinical Medical Research Center For Hereditary Birth Defects, Central South University (Hunan children’s hospital), Hunan Changsha, PR China

**Keywords:** Selenium, Selenoproteins, *EEFSEC* gene, Oxidized lipids, Progressive cerebellar atrophy, Developmental delay

## Abstract

Selenium, an essential micronutrient integrated into selenoproteins as selenocysteine, is fundamental to human health. These selenoproteins are vital for several physiological functions, including maintaining redox balance, safeguarding DNA, and metabolizing thyroid hormones, and are produced via complex pathways involving Sec-tRNA^[Ser]Sec^, the SECIS element, and specific proteins such as eEFSec. This study investigates a 4-year-old girl with global developmental delay and cerebellar atrophy, revealing compound heterozygous variants in the *EEFSEC* gene (p.V488Dfs*113 and p.R443P) through extensive genetic analysis and whole exome sequencing. Both functional prediction tools and structural analysis underscored the detrimental impact of the p.R443P variant. Notably, the patient’s plasma exhibited elevated levels of oxidized fatty acid metabolites compared to those in healthy controls, suggesting an impairment in antioxidant mechanisms. This case link a human disease directly to variants in the *EEFSEC* gene, emphasizing its vital role in cerebellar atrophy and the broader implications for genetic disorders related to defects in selenoprotein synthesis. The results highlight the significance of genetic screening for *EEFSEC* variants in similar cases, potentially broadening the spectrum of known genetic subtypes associated with selenoprotein translation abnormalities.

**Supplementary Information:**

The online version contains supplementary material available at 10.1186/s11689-025-09632-6.

## Introduction

Selenium, an indispensable micronutrient woven into the fabric of life, manifests its biological importance through its incorporation as the amino acid selenocysteine—recognized as the 21 st proteinogenic amino acid [1]. In humans, 25 selenoprotein genes encode a diverse array of selenoproteins, including prominent antioxidant enzymes such as glutathione peroxidase, thioredoxin reductase, and iodothyronine deiodinase [2]. These selenoproteins play a crucial role in maintaining redox homeostasis, protecting DNA and cellular membranes from oxidative damage, and regulating thyroid hormone metabolism [3]. Their roles extend to modulating immune function, gene expression, and protein folding. The substitution of selenocysteine with serine or cysteine can lead to substantial loss or complete abolishment of selenoenzyme catalytic activity [4, 5]. The intricate biosynthesis of selenoproteins involves Sec-tRNA^[Ser]Sec^, the selenocysteine insertion sequence (SECIS) element, and specialized protein factors, which collectively ensure the precise incorporation of selenocysteine at in-frame UGA codons within selenoprotein mRNAs [5, 6].

The biosynthesis of Sec-tRNA^[Ser]Sec^ is a unique process comprising a series of enzymatic reactions. It begins with SerRS-mediated serine activation and attachment to tRNA^[Ser]Sec^. The Ser-tRNA^[Ser]Sec^ is then phosphorylated by O-phosphoseryl-tRNA kinase to form Sep-tRNA^[Ser]Sec^, which is subsequently converted into Sec-tRNA^[Ser]Sec^ by the SEPSECS enzyme in the presence of selenophosphate and pyridoxal phosphate [7–9]. Variants in the *SEPSECS* gene have been implicated in a range of neurological conditions, including intellectual and developmental delays, spasticity, epilepsy, and axonal neuropathy [10]. Additionally, some individuals suffer from autosomal recessive pontocerebellar hypoplasia type 2D (PCH2D), also known as progressive cerebellocerebral atrophy (PCCA), characterized by optic nerve atrophy, hypotonia, progressive microcephaly, and degeneration of cerebellar and cerebral regions [11].

In eukaryotes, the efficient recoding of UGA as Sec necessitates at least two trans-acting factors: SECIS binding protein 2 (SBP2) and the Sec-specific eEFSec [5]. SBP2 specifically identifies and attaches to the SECIS element, a distinctive stem-loop structure located within the 3’ untranslated region (UTR) of selenoprotein mRNA transcripts. This binding action by SBP2 is critical in guiding the ribosome to the mRNA, thereby strategically aligning it with the UGA codon earmarked for selenocysteine insertion. Concurrently, SBP2 engages in a pivotal interaction with eEFSec. Variants in *SECISBP2* can manifest in a range of clinical phenotypes across multiple tissues, with symptoms including developmental delays, hearing loss, fatigue, vertigo, muscle weakness, and SELENON-like deficiency myopathy [12]. Some patients may also experience endocrine alterations, primary infertility, skin photosensitivity, and Raynaud’s phenomenon, with variable symptomatology depending on the affected selenoproteins and tissues [13].

eEFSec is a highly specialized translation elongation factor that exhibits a marked specificity for Sec-tRNA^[Ser]Sec^. Structurally, human eEFSec is comprised of four domains, elegantly organized into a chalice-like conformation; domains 1 to 3 sculpt the cup, reminiscent of the EF-Tu factor, while domain 4 forms the sturdy base [6]. In a finely tuned molecular ballet, SBP2 directs eEFSec, when complexed with Sec-tRNA^[Ser]Sec^, to the ribosomal A site. This precise positioning ensures that the ribosome interprets UGA codons, within the framework of a SECIS element, as cues for selenocysteine insertion, not as termination signals. Upon accurate alignment at the A site, eEFSec facilitates the integration of selenocysteine into the emergent polypeptide [1]. The critical role of eEFSec in selenoprotein biosynthesis is starkly illustrated by the selenoprotein synthesis deficiency observed in *EEFSEC* gene knockout Drosophila [14].

This study reports a child presenting with intellectual disability, dysarthria, singsong speech patterns, and cerebellar atrophy. Serum analysis indicated impaired selenoprotein synthesis and elevated lipid peroxides, which were attributable to compound heterozygous variants in the *EEFSEC* gene.

## Subject and methods

### Participant recruitment and ethical considerations

This study was conducted in accordance with the Declaration of Helsinki and approved by the Institutional Review Board (IRB) of Hunan Provincial Maternal and Child Health Care Hospital. Written informed consent was obtained from the legal guardians of all participants involved in the study. To ensure the protection of our patient’s and control subjects’ privacy, all personal identifiers were removed from the study data.

### Patient and control subject selection

The patient, a 4-year-old girl, was referred to Hunan Provincial Maternal and Child Health Care Hospital due to global developmental delay and weakness. A comprehensive clinical evaluation was performed, including neurological examinations such as language assessment, coordination, nystagmus, muscle strength, reflexes, and muscle tone, as well as development assessment. Blood routine, lipids, lactate, ammonia, thyroid function, muscle enzymes, folic acid, vitamins, amino acid and acylcarnitine spectrum analysis, and other blood biochemistry and urinary organic acid tests were conducted. Additional tests included head magnetic resonance imaging, electroencephalogram (EEG), thyroid ultrasound, and Neostigmine test. For comparative purposes, we also included three age- and sex-matched control subjects. These control subjects were recruited from Hunan Provincial Maternal and Child Health Care Hospital and had no history of abnormal development, no neurological diseases, and no clinical manifestations similar symptoms related to this patient.

### Genetic variant analysis

Whole Exome Analysis (WES). The patient’s and parents’ DNA sample was subjected to exome enrichment using the Nextera Rapid Capture Exome v1.2 kit (Illumina). Sequencing was performed on the HiSeq4000 platform (Illumina), generating 150-bp paired-end reads. Data analysis was conducted using Berry Genomics’ proprietary Verita Trekker Variant Detection System and the Enliven Variant Annotation and Interpretation System, with reference to the human genome assembly hg19 (GRCh37). Variants were excluded if they failed to meet the following quality control criteria: a depth of coverage less than 10, an allele balance below 0.25, or a Phred quality score below 20. In parallel with our investigation, Copy Number Variants sequence (CNV-seq) was conducted adhering to the established protocol to rule out the presence of CNV variations [15].

Mitochondrial DNA Variant Detection. Genomic DNA from participants was fully amplified using specific primers tailored for mitochondrial DNA. The resultant amplified products were subsequently fragmented, culminating in the assembly of a genomic DNA library (NEB#E7370L). Sequencing was performed on an advanced next-generation high-throughput sequencer (Illumina), and the resulting data were meticulously aligned with the mitochondrial genome reference sequence NC_012920.1 from the NCBI database using NextGene V2.3.4 software. This alignment process included a thorough evaluation of both the coverage and sequencing quality of the targeted regions. Variants were selectively filtered according to rigorous criteria and annotated with data derived from the Mitomap database. For pathogenic variants that were conclusively identified (exhibiting a variation frequency exceeding 15%), validation was executed via Sanger sequencing.

### Plasmid construction and cell line generation

Human wild-type *EEFSEC* (NM_021937.5) cDNA was amplified by PCR from a human brain cDNA library and verified by Sanger sequencing. The wild-type *EEFSEC* cDNA was cloned into a pcDNA3.1-3xHA-IRES-EGFP vector. Site-directed mutagenesis using a KOD-Plus-Mutagenesis kit (TOYOBO, Osaka, Japan) was performed to generate two separate mutant constructs: c.1463_1464del (p.V488Dfs*113) and c.1328G > C (p.R443P), in accordance with the manufacturer’s instructions. HEK293 cells were transfected using Lipofectamine 3000 (Invitrogen, USA) according to the manufacturer’s instructions. Transfection efficiency was assessed 48 h post-transfection using fluorescence microscopy and flow cytometry.

### Immunoblotting

Cells were lysed in RIPA buffer (50 mM Tris pH 7.5, 150 mM NaCl, 0.1% SDS, 0.5% sodium deoxycholate, 1% Triton X-100) with protease inhibitors (Roche). Proteins were quantified (BCA assay), separated by SDS-PAGE, and transferred to nitrocellulose membranes (0.45 μm). Membranes were blocked with 5% non-fat milk/TBS-T and probed with Anti-HA (1:1000, #3724, Cell Signaling Technology). After three washes with TBS-T, the membranes were incubated with horseradish peroxidase (HRP)-conjugated secondary antibodies for 1 h at room temperature. Following final washes, protein signals were developed using enhanced chemiluminescence (ECL, Thermo Fisher Scientific) and digitally captured with the ChemiDoc MP Imaging System (Bio-Rad Laboratories). Band intensities were quantified using Fiji software and normalized to GAPDH loading controls.

### Analysis of GSH activity and SELENOF levels by ELISA

Fasting venous blood samples (3–5 mL) were collected from the proband, both parents, and age- and sex-matched healthy controls. The blood samples were allowed to clot at room temperature for 30 min, then centrifuged at 2,000 rpm for 20 min at 4 °C. The supernatant serum was aliquoted into sterile EP tubes and stored at − 80 °C until analysis. Serum glutathione (GSH) activity was quantified using a commercial ELISA kit (Catalogue No. BC012, ELK Biotechnology, China), and serum SELENOF levels were measured using another commercial ELISA kit (Catalogue No. ELK0512, ELK Biotechnology, China), following the manufacturer’s instructions. Absorbance was measured at 412 nm for GSH activity and at 450 nm for SELENOF using a microplate reader (Model Cmax Plus, Molecular Instruments). All samples were analyzed in duplicate.

### Quantitative detection of oxidized fatty acids

Plasma samples were collected from the patient and three control subjects. The samples were then thawed, and 20 µl of the internal standard was diluted in 1 milliliter of ultra-pure water containing formic acid for subsequent enrichment via solid phase extraction (SPE). The SPE cartridge was activated with 1 milliliter of methanol and equilibrated with 1 milliliter of water before the sample application. After loading, the cartridge was washed with 10% methanol and then eluted with 1.2 milliliters of methanol. The eluted fractions were collected, dried, and re-suspended in 40 µl of methanol for UHPLC-MS/MS analysis. The UHPLC-MS/MS was conducted using an Agilent 1290 Infinity I UHPLC system linked to a 6470 A Triple Quadrupole mass spectrometer (Santa Clara, CA, USA). Data acquisition was facilitated through dynamic multiple reaction monitoring (MRM) in optimized MRM transitions, with the MassHunter software (version B.08.00, Agilent) managing instrument control and data collection.

### Structural analysis

The impact of variants on protein spatial structure was predicted using Alphafold2 (version 2.3.2) with default parameters, as described by Jumper et al. [16] Alphafold2 leverages deep learning algorithms to predict protein structures with high accuracy, providing insights into potential conformational changes induced by genetic variants. The predicted structures were further analyzed and visualized using PyMOL software (version 3.0) to examine the spatial arrangement of amino acids and assess the potential functional consequences of the variants. This approach allowed us to evaluate how specific mutations might alter protein stability, interactions, or functional domains.

### Statistical analysis

The data are presented as mean ± standard deviation (SD). Statistical analyses and figure generation were performed using R software (version 4.3.2). Comparisons of expression levels between the two mutant proteins and the wild-type protein were conducted using unpaired T-test, with a p-value < 0.05 considered statistically significant. Serum selenoprotein levels and plasma lipid peroxidation levels were evaluated using a reference range-based assessment method derived from control group distributions.

## Results

### Patient’s clinical presentation

The patient is a female child born to non-consanguineous parents, with a normal prenatal history and no congenital anomalies at birth. There is no special family history. A marked delay was noted in her gross motor skill development; she mastered the ability to independently turn over at the age of 7 months. Initial diagnostic evaluations, encompassing a head MRI and a 24-hour EEG, did not identify any notable abnormalities. Laboratory tests identified elevated plasma total cholesterol levels at 5.6 nmol/L, although other critical parameters such as lactic acid and blood ammonia were within normal ranges. Subsequent clinical follow-ups indicated a noticeable delay in achieving motor developmental milestones relative to her peers, while her language acquisition and cognitive development seemed to be less impacted. Language development delays were evidenced by her ability to say “baba, mama” only by 32 months, and by 36 months, she could recite simple nursery rhymes, albeit with clear articulation issues. Blood tests repeatedly observed high plasma cholesterol levels.

**Fig. 1:**
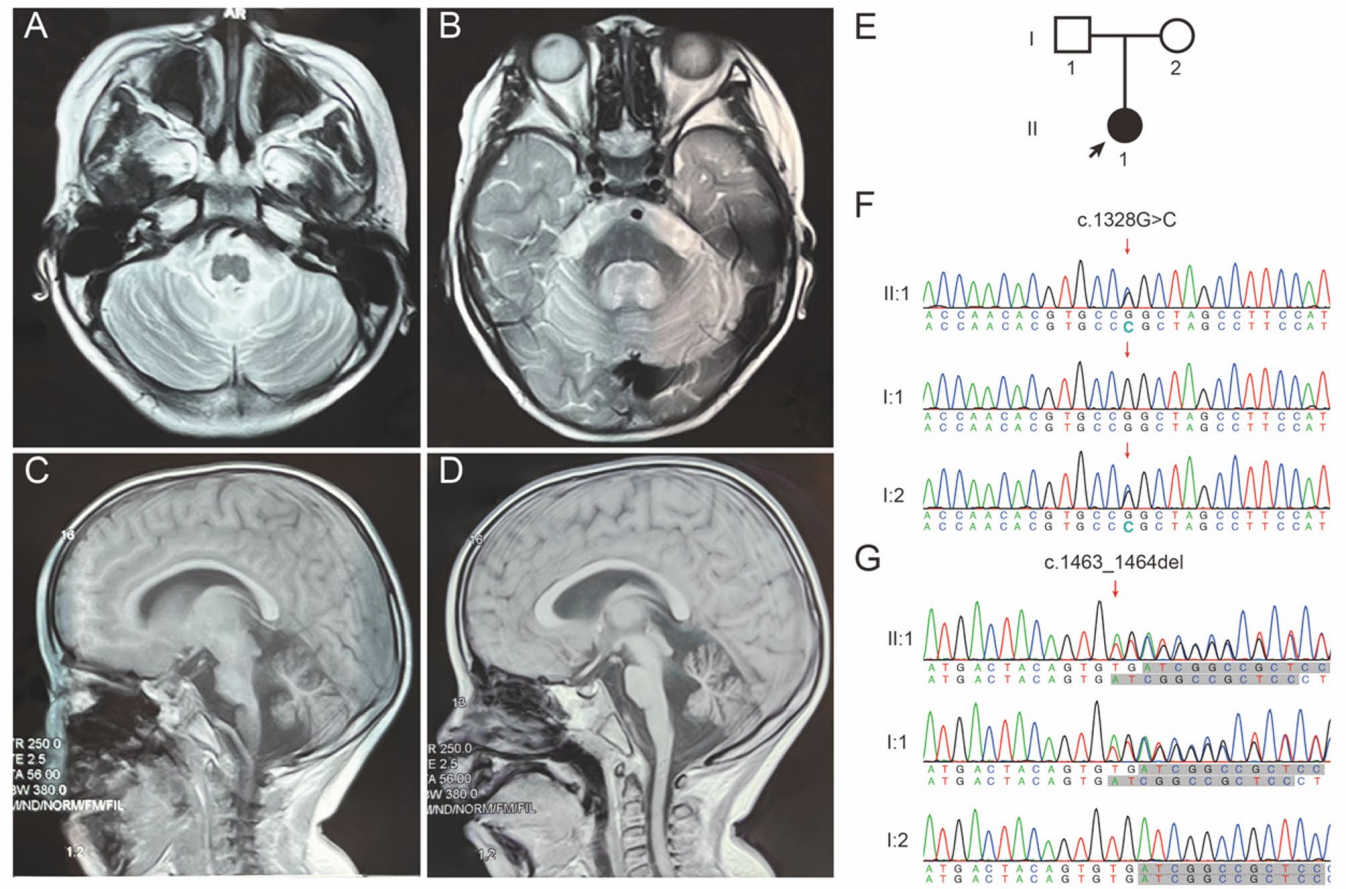
Neuroimaging Findings and Genetic Sequencing Results of the Proband. **A**-**D** Progressive cerebellar atrophy demonstrated by serial MRI scans (sagittal and axial views). **E** Pedigree chart showing the familial inheritance pattern. **F**-**G** Sanger sequencing chromatograms of the *EEFSEC* gene variants: (**F**) Missense mutation (c.1328G > C, p.R443P) and (**G**) Frameshift mutation (c.1463_1464del, p.V488Dfs*113), with arrowheads indicating the variant loci

Currently, at the age of four, she is unable to sit or stand without assistance and exhibits dysarthria with a notable “sing-song” speech pattern, and water aspiration. Moreover, she encounters difficulties in language expression, being unable to form complete sentences to effectively communicate her needs. Nevertheless, she can comprehend and act on straightforward directives and recognizes familiar objects. She is in poor spirits and, neurological examinations have revealed ankle clonus and hyperactive tendon reflexes; her alternating movements are slow. However, she does not exhibit nystagmus, limb tremors, or intention tremors, and evaluations of her limb muscle tone are within normal limits. Recent head MRI scans have shown atrophy of the cerebellar hemispheres and ventricular dilation (Fig. 1A and D).

### Genetic testing results

WES identified a heterozygous frameshift variant in the *EEFSEC* gene (NC_000003.11(NM_021937.5):c.1463_1464del, p.V488Dfs113) and a heterozygous missense variant (NC_000003.11(NM_021937.5):c.1328G > C, p.R443P) in the proband (II:1). The I:1 (father of proband) possesses the heterozygous variant p.V488Dfs113 but not p.R443P, while the I:2 (mother of proband) carries the heterozygous variant p.R443P without the p.V488Dfs*113 variant (Fig. 1E, F and G). Neither variant has been previously reported in the databases referenced in the study’s methodology. These findings were corroborated by subsequent Sanger sequencing. No additional variants that could account for the patient’s clinical symptoms were identified, including those in the mitochondrial genome or potential CNVs.

**Fig. 2:**
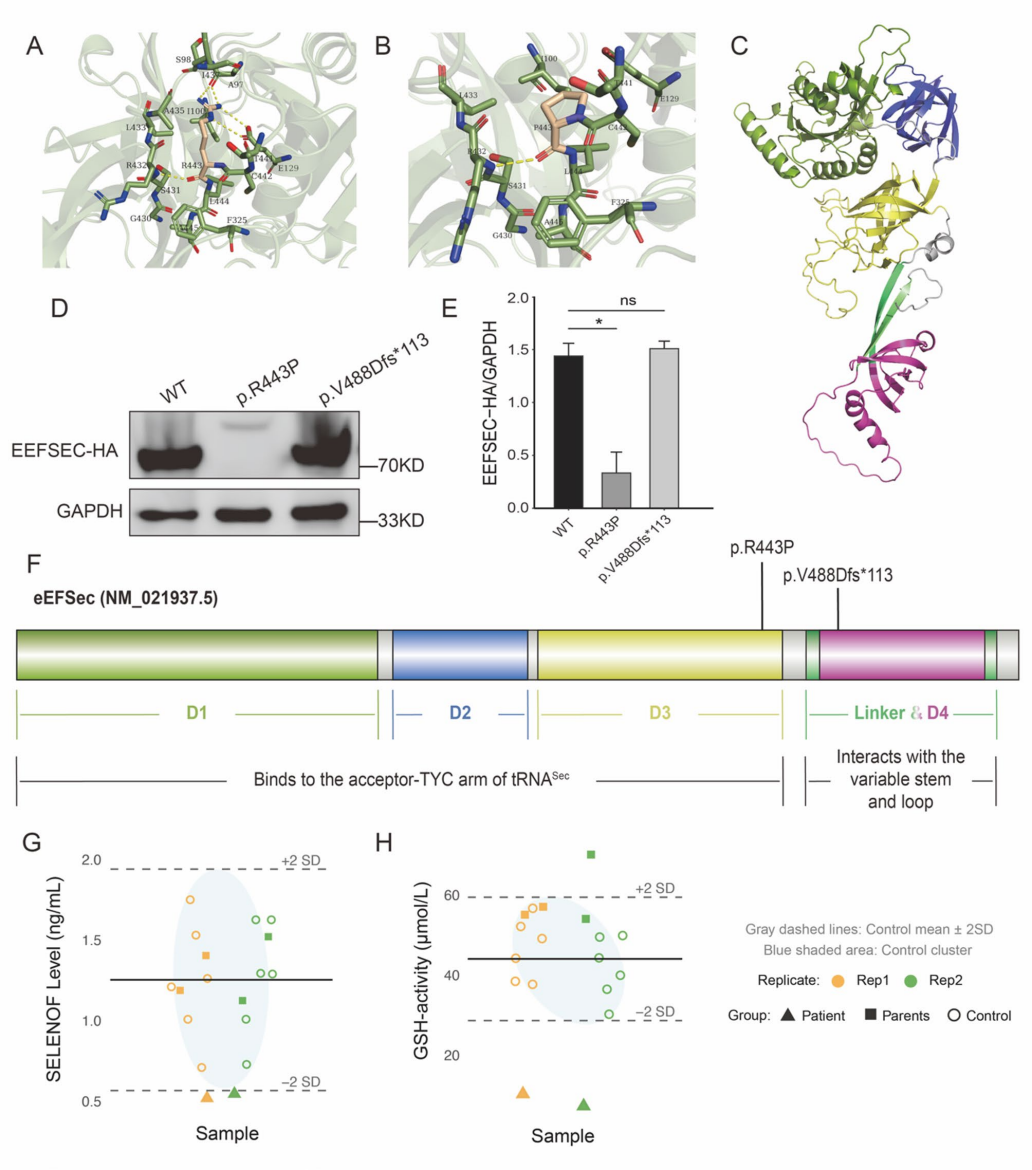
Structural and Functional Characterization of eEFSec Mutants and Serum Selenoprotein Analysis. **A**–**B** Predicted structural impact of the R443P variant. **A** Wild-type R443 (wheat) forms multiple hydrogen bonds (yellow dashes) with neighboring residues (gray). **B** Mutant P443 (wheat) disrupts these interactions, reducing local stability. **C** Overall 3D structure of eEFSec, with mutated domains highlighted. **D**–**E** eEFSec protein expression in patient-derived cells, showing significant reduction (*p* < 0.05). **F** Domain architecture of eEFSec with variant locations. **G**–**H** Serum biomarkers: (**G**) SELENOF expression levels and (**H**) glutathione peroxidase (GSH) activity in patients versus controls

Analysis with 22 functional prediction tools (Supplementary Table), such as SIFT, Polyphen-2, and CADD, indicated that the p.R443P variant is deleterious. Structural analysis using Alphafold2 and PyMOL revealed that the hydrogen bonding around the amino acid residue at position 443 was altered by the variant; the mutated residue retained only a hydrogen bond with residue R432, losing all other hydrogen bonds with nearby residues (A97, E129, T441). Furthermore, the number of residues within 5 Å of the mutated residue decreased from 15 to 11, suggesting a potential impact on the protein’s structural stability (Fig. 2A and B).

### Protein expression analysis of *EEFSEC* variants

To investigate whether the variants affect eEFSec protein expression, we generated HA-tagged expression constructs and conducted Western blot analysis in HEK293 cells. Notably, the p.R443P variant demonstrated markedly reduced protein expression relative to wild-type eEFSec, along with altered electrophoretic mobility (Fig. 2D and E), indicative of potential conformational alterations or post-translational modifications. In contrast, the p.V488Dfs*113 variant exhibited expression levels similar to wild-type (Fig. 2D and E), presumably because the minimal length difference (599 aa versus 596 aa) allowed it to evade nonsense-mediated mRNA decay (NMD). Structural modeling further demonstrated that although the p.V488Dfs*113 variant maintained comparable expression levels, it would truncate the majority of the D4 and Linker domains (Fig. 2C and F), which are critical structural elements required for eEFSec’s biological function.

### Serum Selenoprotein levels in the patient

eEFSec is critical for selenoprotein biosynthesis. To assess whether the reduction of eEFSec affects the patient’s efficiency in synthesizing selenoproteins, we measured the patient’s serum GSH activity and SELENOF concentration. The results showed that the patient had substantially lower serum selenoprotein levels compared to healthy controls. The mean GSH activity was 9.347 µmol/L, which was below the − 2SD threshold of control values (44.59 ± 7.62 µmol/L). Similarly, the mean SELENOF concentration was 0.54 ng/mL, significantly lower than the control − 2SD level (1.27 ± 0.36 ng/mL). These findings demonstrate a selective selenoprotein deficiency in the proband.

### Plasma lipid peroxidation levels results

Previous research has highlighted an increase in plasma lipid peroxidation products in individuals with disorders of selenoprotein synthesis. In our study, we aimed to ascertain if a patient with an *EEFSEC* gene variant exhibits comparable alterations. We assessed the plasma levels of oxidized fatty acid metabolites, including those derived from arachidonic and linoleic acids, by employing mass spectrometry. Our analysis included a comparison with data from gender- and age-matched healthy controls (Table 1). The findings revealed that the patient’s plasma contained markedly higher levels of oxidized metabolites, such as 15-Keto-PGE2, 11(12)-DiHET, 13-HpODE, 9,10-DiHOME, 9-HODE, and 9-HpODE (Table 1 and Supplementary Table). However, certain indicators in the patient, such as 5(6)-DiHET (an ω−6 arachidonic acid metabolite), 16(17)-DiHDPA (a DPA derivative), and HDHA/HEPE species (ω−3 DHA/EPA metabolites), were reduced compared to the control group.

**Table 1:** Quantitative results of plasma oxidized fatty acids increased in the patient compared to the control group

		Control 1	Control 2	Control 3	Control means	Patient
General Information	Sex	Female	Female	Female	-	Female
	Age	4y2m	4y1m	3y11m	-	4y0m
Metabolite	Patient Changes					
15-Keto-PGE2	Up	0.063	0.059	0.068	0.063 ± 0.005	0.090
11(12)-DiHET	Up	0.814	0.700	0.746	0.753 ± 0.058	1.029
13-HpODE	Up	0	0	0.513	0.171 ± 0.296	0.809
9,10-DiHOME	Up	0.883	2.514	2.242	1.879 ± 0.834	4.605
9-HODE	Up	4.325	2.373	3.200	3.299 ± 0.981	5.740
9-HpODE	Up	0.000	0.000	0.000	0.000	0.828
5(6)-DiHET	Down	0.264	0.217	0.186	0.223 ± 0.039	0.077
16(17)-DiHDPA	Down	0.638	0.645	0.653	0.646 ± 0.007	0.364
14-HDHA	Down	3.935	4.569	5.916	4.806 ± 1.012	1.770
17-HDHA	Down	0.355	0.370	0.488	0.404 ± 0.073	0.201
13-HDHA	Down	0.242	0.268	0.274	0.261 ± 0.017	0.159
16-HDHA	Down	0.169	0.141	0.188	0.166 ± 0.024	0.088
12-HEPE	Down	1.158	0.927	1.235	1.107 ± 0.161	0.461

*15-Keto-PGE2* 15-Keto Prostaglandin E2, *11(12)-DiHET* 11,12-Dihydroxy-5Z,8Z,14Z-eicosatrienoic acid, *13-HpODE* 13-Hydroperoxy-9Z,11E-octadecadienoic acid, *9,10-DiHOME* 9,10-Dihydroxy-12Z-octadecenoic acid, *9-HODE* 9-Hydroxy-10E,12Z-octadecadienoic acid, *9-HpODE* 9-Hydroperoxy-10E,12Z-octadecadienoic acid, *16(17)-DiHDPA* 16,17-Dihydroxy-4Z,7Z,10Z,13Z,19Z-docosapentaenoic acid, *14-HDHA* 14-Hydroxy-4Z,7Z,10Z,12E,16Z,19Z-docosahexaenoic acid, *17-HDHA* 17-Hydroxy-4Z,7Z,10Z,13Z,15E,19Z-docosahexaenoic acid, *13-HDHA* 13-Hydroxy-4Z,7Z,10Z,14E,16Z,19Z-docosahexaenoic acid, *16-HDHA* 16-Hydroxy-4Z,7Z,10Z,13Z,17E,19Z-docosahexaenoic acid, *12-HEPE* 12-Hydroxy-5Z,8Z,10E,14Z,17Z-eicosapentaenoic acid.

The measurement unit is ng/ml.

## Discussion

Our study introduces the first documented case of a child exhibiting intellectual disability, dysarthria characterized by a unique singsong speech pattern, cerebellar atrophy, and consistently elevated total plasma cholesterol levels. WES has identified that she carries compound heterozygous variants in the *EEFSEC* gene. Our findings are consistent with extensive genetic research that underscores the essential role of selenoprotein synthesis in human health and development. The clinical manifestations observed in this patient offer unprecedented insights into the phenotypic range related to disturbances in selenoprotein biosynthesis, with a specific emphasis on the involvement of the *EEFSEC* gene.

The synthesis of selenoproteins is a complex, multi-step process essential for the proper integration and functionality of selenium, an indispensable micronutrient, in its selenocysteine form (Sec) [17]. This form is acknowledged as the 21 st proteinogenic amino acid. The emergence of developmental delays, neurological impairments, and oxidative stress due to variants in this biosynthesis pathway’s components highlights the critical nature of these processes [17, 18]. As our patient exhibits compromised development potentially linked to *EEFSEC* gene variants, eEFSec protein has been shown to specifically bind the Sec-tRNA[Ser]Sec. It is hypothesized to operate autonomously from a guanine nucleotide exchange factor, owing to its high intrinsic GTP affinity [5, 6]. Consequently, the *EEFSEC* gene plays a vital role in decoding UGA codons as selenocysteine, rather than as termination signals.

The protein expression assay revealed a significant reduction in the expression level of the R443P variant compared to the wild-type. Human eEFSec protein is structured into four domains (D1-D4), creating a chalice-like formation. Detailed analysis of the D3 domain uncovers multiple loop insertions prone to solvent exposure, notably between β17 and β18 (residues 352–373), β21 and β22 (residues 432–444), and β18 and β19 (residues 378–410). The R443P missense variant is situated within the D3 domain, a region responsible for maintaining eEFSec protein stability. Research indicates that the D4 domain’s orientation is solidified through interactions between the loop β28-α11 at the extreme C-terminus and D3 residues. The formation of a hydrogen bond between Glu372 in D3 and Lys582 in the C-terminal segment is particularly pivotal [6]. The K582A and 582KRYVF586 -> AAAAA variants show reduced expression levels and stability compared to wild-type eEFSec [6]. This underscores the significance of amino acid residue hydrogen bonds in maintaining eEFSec’s structural integrity. We therefore propose that the R443P variant diminishes amino acid interactions at position 443 and disrupts key hydrogen bonds, potentially destabilizing eEFSec’s chalice-like structure. This structural perturbation likely reduces eEFSec stability, ultimately impairing its functional activity. On the other hand, although the V488Dfs*113 variant does not exhibit significant changes in protein expression, it induces structural alterations in the D4 and Linker domains. Previous studies have shown that these regions are critical for binding to the variable stem and loop of tRNA^Sec^, while the D4 domain specifically interacts with the apical loop of SECIS — both essential for UAG codon decoding. Variants or deletions in D4 have been demonstrated to severely impair eEFSec activity and selenoprotein synthesis. Therefore, the V488Dfs*113 variant is likely to disrupt eEFSec function. The decreased SELENOF levels and GSH activity in our patients support the conclusion that the *EEFSEC* compound heterozygous mutations impair eEFSec’s efficiency in translating the UGA codon, thereby hindering adequate selenoprotein synthesis.

Selenoproteins are known for their antioxidant properties, effectively neutralizing reactive oxygen species (ROS) [18]. While ROS play a crucial role in various biological functions such as cellular communication, growth modulation, oncogenesis inhibition, and immune defense at moderate levels, an overabundance can lead to oxidative stress, causing harmful alterations to DNA, proteins, and lipids [19, 20]. This, in turn, triggers a range of diseases, particularly those associated with neuronal degeneration. Our study expands the knowledge of lipid peroxidation products in the context of disorders of selenoprotein synthesis. Increased levels of oxidized fatty acid metabolites in the patient’s plasma, compared to healthy individuals, shed light on an aspect of oxidative stress potentially pivotal in the pathogenesis of conditions related to *EEFSEC* variants. This finding is consistent with previous research on an individual with variants in the *SBP2* gene [21] and offers a biochemical basis for the phenotypic symptoms observed in our patient. Although we detected an increase in the patient’s cholesterol levels, we were unable to measure 7β-hydroxycholesterol, which might better explain the observed rise in cholesterol. Additionally, the observed reductions in multiple oxylipins, including 5(6)-DiHET, 16(17)-DiHDPA, and some HDHA/HEPE species, suggest a broad suppression of polyunsaturated fatty acid (PUFA) metabolic pathways. This systemic alteration in lipid mediator networks may potentially impact neurodevelopment [22, 23], although the precise relationship between PUFA metabolism and selenoprotein pathways requires further elucidation. The underlying mechanisms driving these metabolic changes remain to be fully investigated.

SEPSECS, which encodes the O-phosphoseryl-tRNA: selenocysteinyl-tRNA synthase, is upstream in the selenoprotein synthesis pathway where eEFSec is located and has been associated with PCCA [10, 11]. Patients compound heterozygous for *SEPSECS* variants c.974 C > G (p.Thr325Ser) and c.1287 C > A (p.Tyr429*) exhibit reduced levels of selenocysteinyl-tRNA in brain tissue and decreased expression of three selenoproteins: thioredoxin reductase TXNRD1 and glutathione peroxidases GPX1 and GPX4 [10]. This finding parallels the observed reduction in GSH activity in our patient. Patients with *SEPSECS* variants typically present with neonatal irritability, spastic or dystonic quadriplegia, virtually absent psychomotor development, axonal neuropathy, and elevated blood/CSF lactate. Neuropathological examination reveals laminar necrosis, severe myelin loss, neuron depletion, and astrogliosis. In contrast, our patient’s primary features are motor developmental delay, inability to sit or walk independently, dysarthria, normal muscle tone, positive pathological signs, low mood, lack of vitality, normal blood lactate levels but recurrently elevated total cholesterol. A common finding in imaging studies is cerebellar atrophy [10]. Whether due to a synthesis block of selenocysteinyl-tRNA caused by *SEPSECS* gene variants or a translation impediment of selenoprotein UGA codon caused by *EEFSEC* gene variants, both lead to cerebellar atrophy in patients, underscoring the importance of selenoproteins in maintaining cerebellar function. Additionally, the elevated oxidative stress markers in our patient’s plasma suggest that cerebellar atrophy may be a consequence of an imbalance in oxidative stress.

In fact, disorders in selenoprotein synthesis can lead to the involvement of multiple systems and present with diverse symptoms. For instance, variants in the *SBP2* gene, which is also involved in the translation of the selenoprotein UGA codon, can lead to characteristic clinical manifestations and the implications of growth hormone (GH) and triiodothyronine in longitudinal bone growth and maturation [24, 25], cutaneous photosensitivity and enhanced UV-mediated oxidative damage, azoospermia with spermatogenic maturation arrest, axial muscular dystrophy similar to SEPN1-related myopathies, impaired T cell proliferation and abnormal cytokine production, increased fat mass but enhanced insulin sensitivity, and hearing loss [12]. However, our patient’s thyroid function is normal, and to date, there has been no detection of photosensitivity, immune disorders, or hearing loss, highlighting the clinical phenotype complexity associated with selenoprotein-related diseases. Moreover, certain genetic polymorphisms in the *EEFSEC* gene have been identified as potential risk factors for various diseases, including chronic obstructive pulmonary disease [26], preterm birth [27, 28], and periodontitis [29, 30]. However, no increased susceptibility to these conditions was observed in either the patient or their family members. This phenomenon may be attributed to variations in selenium-protein deficiency susceptibility stemming from the patient’s unique environmental factors and genetic background.

Given the essential role of selenium and selenoproteins in various biological processes, variants in genes involved in selenoprotein biosynthesis, such as eEFSec, can have profound implications. These implications extend beyond oxidative stress to include neurological development [31, 32], immunity [33–35], and thyroid hormone metabolism [25], among others. Consequently, there’s a pressing need for in-depth research into the pathophysiological mechanisms of disorders tied to selenoprotein synthesis, particularly concerning the *EEFSEC* gene. Such research could provide critical insights into developing targeted therapeutic strategies for affected individuals.

In summary, we report a child with progressive cerebellar atrophy caused by variants in the *EEFSEC* gene and confirm its role in the pathogenesis of the disease in the reported case. Our findings emphasize the importance of considering *EEFSEC* gene variants in patients presenting with similar clinical spectrums. Progressive cerebellar atrophy due to a compound heterozygous pathogenic variant in the *EEFSEC* gene expands the genetic subtypes of this disease. Variants in genes involved in selenoprotein translation, such as *SEPSECS* and *EEFSEC*, can lead to progressive cerebellar atrophy, demonstrating the vital role of selenoproteins in maintaining cerebellar function.

## Supplementary Information

Supplementary Material 1.

## Data Availability

No datasets were generated or analysed during the current study.
